# Metabolic Phenotyping Identifies Distinct Urinary Anti-Adhesion Phenotypes and Differential Responses to Cranberry Juice Intake in Healthy Women

**DOI:** 10.3390/nu18193250

**Published:** 2026-10-01

**Authors:** Shuhan Li, Manasi Kamat, Diana H. Taft, Timothy J. Garrett, Liwei Gu

**Affiliations:** 1 Food Science and Human Nutrition Department, Institute of Food and Agricultural Sciences, University of Florida, Gainesville, FL 32611, USA; 2 SECIM-CTSI, College of Medicine, University of Florida, Gainesville, FL 32611, USA; 3 Department of Pathology, Immunology and Laboratory Medicine, College of Medicine, University of Florida, Gainesville, FL 32611, USA

**Keywords:** cranberry, urinary tract infections, Uropathogenic *E. coli*, metabolomics

## Abstract

**Background**: Cranberry products have been used by millions of women to prevent urinary tract infections (UTIs), although clinical trials have yielded inconsistent results. **Objectives**: This phenotyping trial aims to test the hypothesis that women’s urine differs in its anti-adhesion capabilities against P-type *Escherichia coli* (UPEC), potentially influenced by variations in their urine metabolome, and that women’s urinary anti-adhesion activity responds differently to cranberry juice intake. **Methods**: A total of 152 healthy women (aged 21–65) avoided any cranberry products for 10 days and consumed 10 oz of pure cranberry juice daily for 4 days. Two baseline and one post-consumption morning urine samples were collected on days 9, 10, and 15, respectively, to assess urinary anti-adhesion capacity using an optimized fluorometric method, and the results were expressed as anti-adhesion indices (AAI). Metabolites in 18 baseline urine samples with high AAI (133–164) and 18 baseline urine samples with low AAI (80–128) were explored using untargeted metabolomics on UHPLC-Orbitrap-MS/MS. **Results**: The baseline urinary AAI against P-type UPEC ranged from 84 to 170 in the women. Seventy-seven women (51%) were assigned to the “UTI-susceptible” phenotype due to a baseline AAI below the 50th percentile (AAI = 131). The rest (*n* = 75) were “UTI-nonsusceptible.” The metabolome differed in urine with high or low AAI, and 165 urinary metabolites were found to differentiate between UTI-susceptible and nonsusceptible phenotypes. After four days of cranberry juice consumption, 34.2% of susceptible women and 18.9% of nonsusceptible women were assigned to the “positive responders” phenotype due to positive AAI changes above 10. The rest were “non-positive responders.” **Conclusions**: Healthy women differed in their urinary anti-adhesion capacities against UTIs and responded differently to cranberry juice consumption. This study suggests that women need personalized recommendations on whether to use cranberry products to prevent UTIs based on their individual metabolic phenotype.

## 1. Introduction

American cranberries are consumed by millions of women to prevent the occurrence or recurrence of urinary tract infections (UTIs), which affect 60% of women in their lifetime and 10% of women annually [1,2]. Asymptomatic bacteriuria in women is diagnosed by the presence of bacteria (mostly UPEC) in urine at a concentration of 10^5^ CFU/mL in two consecutive specimens. Symptomatic patients with acute cystitis (bladder infection) or pyelonephritis (kidney infection) are diagnosed when urine contains ≥ 10^3^ or ≥10^4^ CFU/mL of uropathogens, respectively. Over 90% of diagnosed UTIs are caused by pathogenic *E. coli* expressing type P or 1 fimbriae. UTIs are initiated by the adhesion of UPEC to the urinary tract epithelia, followed by invasion and the formation of intracellular bacterial communities [3]. After initial diagnosis and treatment with antibiotics, about 30–44% of women will have a recurrence within three months. About 20–50% of women will experience multiple recurrences within a year [4].

Uropathogenic *E. coli.* P fimbriae adhere to the glycosphingolipids on the surface of urinary epithelia. They are the primary initiator of UTI because they mainly adhere to epithelial cells, whereas type 1 fimbriae adhere to each other [5]. P fimbriae are expressed by up to 100% of strains causing acute pyelonephritis and urosepsis in otherwise healthy individuals [6].

Consumption of cranberry juice cocktail (27% juice) enhanced the anti-adhesion activity of human urine based on a hemagglutination assay specific for P-fimbriated UPEC. Other foods, including apple juice, grape juice, tea, and dark chocolate, did not have such activity. The unique bioactivity of cranberry juice was largely attributed to its A-type procyanidin content [7]. In 2023, a large-scale meta-analysis in The Cochrane Database of Systematic Reviews analyzed 50 clinical trials on 8857 randomized participants. The authors noted that the results were inconsistent due to high heterogeneity and imprecise due to wide confidence intervals. The certainty of evidence was rated from low to moderate for cranberries’ ability to reduce the risk of UTIs in women [8]. For example, a randomized, double-blind, placebo-controlled trial showed that one cup of cranberry juice daily for 24 weeks lowered the number of UTI episodes in 185 women with a recent history of UTIs [9]. But in a similar trial, one cup of cranberry juice twice daily for 6 months failed to prevent UTI recurrence in 319 women with a history of acute UTIs [10]. Conflicting results were also observed in two double-blinded, placebo-controlled trials in which women received cranberry extracts providing over 36 mg of proanthocyanins daily for 12 weeks or 360 days, respectively [11,12].

These variations raise doubts about the efficacy of cranberries for UTI prevention, posing significant challenges for clinicians seeking to use the literature to guide prevention strategies [13]. It is unknown whether inter-individual differences in intrinsic urinary anti-adhesion capacity and metabolomic profiles contribute to variable intervention responses. We hypothesize that human urine differs in its inherent anti-adhesion capabilities against P-type UPEC, potentially influenced by variations in its urine metabolome, and that women’s urinary anti-adhesion activity responds differently to cranberry juice intake. The hypothesis is based on the concept that metabolic phenotyping of interindividual variability in human physiology and dietary responses is pivotal to personalized nutrition, which recognizes that nutritional needs and responses vary among individuals rather than adopting a one-size-fits-all approach.

The primary objective of this research was to investigate and phenotype the inherent anti-adhesion activity in the urine of healthy women and its changes after drinking cranberry juice. An exploratory objective is to assess the urine metabolome in women with low or high inherent anti-adhesion capacity. The purpose of this screening trial is to classify participants into phenotypes, if any exist, to prepare for a randomized controlled trial.

## 2. Materials and Methods

### 2.1. Materials and Chemicals

Creatinine was purchased from MilliporeSigma (St. Louis, MO, USA). Human uroepithelial HTB-4 T24 cells, Eagle’s Minimum Essential Medium, and P-type fimbriated *E. coli* strain CFT073 were purchased from American Type Culture Collection (Manassas, VA, USA). Cyanidin-3-galactoside, peonidin-3-glucoside, (-)-epicatechin, caffeic acid, myricetin, quercetin, kaempferol, formic acid, acetonitrile, methanol, anhydrous ethanol, 4-dimethylaminocinnamaldehyde (DMAC), sodium azide, and synthetic human urine were purchased from Thermo Fisher Scientific Inc. (Waltham, MA, USA). The synthetic human urine consisted of sodium chloride (<1%), urea (1–3%), magnesium sulfate heptahydrate (<0.1%), calcium chloride dihydrate (<0.1%), and water (>95%).

### 2.2. Study Beverage and Polyphenol Analysis

Unsweetened pure cranberry juice produced by an American agricultural cooperative was used as an intervention in the study. Anthocyanins, flavonols (myricetin, quercetin, and kaempferol), and caffeic acid in cranberry juice were analyzed on HPLC using published methods [14]. Standard curves of anthocyanidins were based on 0–62.5 µg/mL cyanidin-3-galactoside and peonidin-3-glucoside. Samples were filtered through 0.45 μm filter units before the analysis.

A DMAC colorimetric assay was conducted to determine the concentration of total procyanidins in cranberry juice using a published method [15]. Cranberry juice was diluted in 91% ethanol. DMAC (0.05 g) was weighed and added to 50 mL of acidified 91% ethanol. To measure the procyanidin concentration, 70 µL of 91% ethanol (blank) and diluted samples were incubated with 210 µL of 0.1% (*w*/*v*) DMAC solution at room temperature for 30 min. The standard curve was based on 0–50 µg/mL (-)-epicatechin. Absorption at 640 nm was measured on a microplate reader.

### 2.3. Outcomes

The primary outcome is the variation in urinary anti-adhesion activity against UPEC in women and their responses to four days of cranberry juice intake. The exploratory outcome was the metabolomic difference in urine between high- and low-anti-adhesion activities.

### 2.4. Study Design

This human study was a screening trial approved by the Institutional Review Board at the University of Florida (IRB202102165, approval date: 13 August 2021) and registered on ClinicalTrials.gov as NCT05065008 (registration date: 29 September 2021). The study was completed at the University of Florida. All the participants signed a consent form before the study.

**Figure 1:**
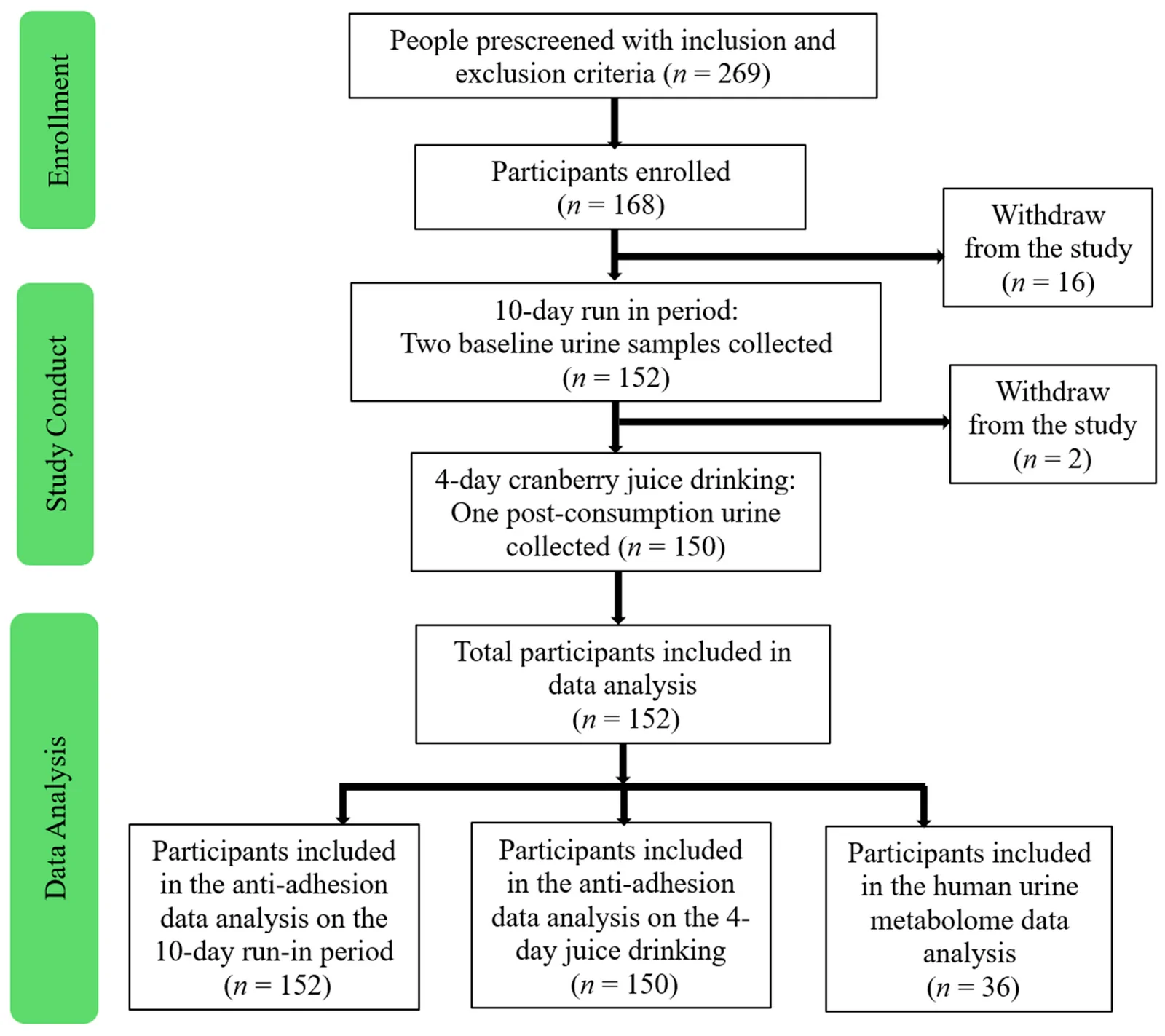
Flow diagram of the metabolic phenotyping trial. The 36 participants in the urine metabolomics analysis include 18 with high baseline AAI and 18 with low baseline AAI.

The experimental design is depicted in Figure 1. Inclusion criteria included age 21–65, BMI 18.5–29.9 kg/m^2^, and at least 110 pounds. Exclusion criteria included BMI ≥ 30 kg/m^2^, pregnancy, breastfeeding, smoking, frequent alcohol use, current UTI, a history of any clinically important disorder that may interfere with the interpretation of the results, and medication use that might affect study outcomes. A total of 152 healthy females aged between 21 and 65 were recruited. The sample size was determined by power analysis (α = 0.05, β = 0.20) for a two-sample t-test, assuming a moderate effect size (Cohen’s d = 0.5) [16].

The study was initiated with a 10-day run-in period. Participants were asked to refrain from any cranberry products, more than 18 mL of alcohol per day, more than two cups of coffee or tea per day, polyphenol-rich foods (blueberries, dark chocolate, cinnamon, red wine, plums, and blackberries), or dietary supplements (isoflavone, resveratrol, grape, pine bark, green tea, or cinnamon extracts). The dietary limits were maintained throughout the trial. Two self-collected baseline urine samples were provided by participants on the mornings of days 9 and 10. The participants then drank 10 oz of cranberry juice daily for 4 days and provided a post-consumption urine sample on the morning of day 5. Participant compliance was assessed *via* emails and phone calls. Participants were asked to return the bottle caps on their last lab visit. Two participants withdrew from the study during the 4-day juice drinking period. All 152 participants finished baseline urine collection, and 150 women completed post-consumption collection. All urine samples were transferred into 15 mL centrifuge tubes and stored at −80 °C prior to use.

### 2.5. Urine Sample Standardization

Ten mL urine samples were freeze-dried and reconstituted in deionized water. Creatinine concentrations in reconstituted urine were measured using a creatinine kit (Thermo Fisher Scientific, Waltham, MA, USA). All urine samples were diluted to a standardized creatinine concentration of 40 mg/dL before analysis for anti-adhesion activities and metabolomics.

### 2.6. Uroepithelial Cell and P-Type Uropathogenic E. Coli Culture

T24 cells were cultured in Eagle’s Minimum Essential Medium supplemented with 10% fetal bovine serum (Fisher Scientific, Pittsburgh, PA, USA) and 1% penicillin-streptomycin solution (MilliporeSigma, St. Louis, MO, USA) at 37 °C, 5% CO_2_ in a 75 cm^2^ cell flask for 3–4 days. Cells grown to confluence were detached with trypsin-EDTA (Fisher Scientific, Pittsburgh, PA, USA). Cell density was measured using a hemocytometer. Cells were diluted in culture medium and transferred to a black 96-well microplate at 10^5^ cells/well, then incubated overnight at 37 °C in 5% CO_2_. Before anti-adhesion assays, cells were washed with PBS, fixed with 100% methanol for 2 min, and dried in a laminar flow hood for use.

*E. coli* strain CFT073 was incubated in 75 mL of tryptic soy broth (VWR International, Radnor, PA, USA) and maintained in log-phase growth for about 24 h. Then, 200 µL of broth with *E. coli* was transferred to colony-forming agar (2% agar with 1% casamino acids, 0.75% yeast extract, 21 mM MgSO_4_·7H_2_O, and 20 mM MnCl_2_·4H_2_O) and incubated at 37 °C overnight to promote the expression of P-type fimbriae.

### 2.7. Anti-Adhesion Assay

A published fluorometric method was modified and applied to evaluate urinary anti-adhesion capacity against P-type UPEC [17,18]. A synthetic human urine with 40 mg creatinine/dL was used as the blank in the assay. P-type *E. coli* CFT073 colonies in agar medium were transferred into PBS and diluted to 0.80 AU at OD_600_ to provide a concentration of approximately 1 × 10^9^ colony-forming units (CFU)/mL. *E. coli* was incubated with 800 μM BacLight Green for 30 min at room temperature in the dark, with a ratio of 1 μL BacLight Green/1 mL *E. coli*-PBS suspension. Labeled *E. coli* was centrifuged at 5000× *g* for 5 min to remove excess BacLight Green, then resuspended in PBS to a final density of 4.6 × 10^8^ CFU/mL. Labeled *E. coli* (100 μL per well) was mixed with 207 μL of standardized human urine samples and the blank in a black 96-well microplate and preincubated for 30 min at 37 °C. After preincubation, the mixture (267 μL per well) was pipetted into another black 96-well microplate containing 10^5^ T24 cells/well to achieve an *E. coli*: T24 cell ratio of 400:1. The plate was incubated for 1 h at 37 °C. Unadhered *E. coli* were removed by PBS washing after incubation. The same procedure was also conducted on unlabeled *E. coli* at the same concentration on the same 96-well plate. The plate was read on the microplate reader with 480 nm excitation/516 nm emission. BacLight Green at 0.25 μM was set to a maximum fluorescence of 80,000 and used as a control to limit the sample fluorescence range. Sample fluorescence values were obtained by subtracting unlabeled *E. coli* fluorescence from labeled *E. coli*. Triplicate samples were used for each sample in the anti-adhesion assays. The intra- and inter-assay coefficients of variation for this anti-adhesion assay were ≤10%.

### 2.8. Anti-Adhesion Index Calculation and Classification Thresholds

The adhesion inhibitions of urine samples were calculated using Equation (1).

*Adhesion inhibition* = 100 × (1 − *sample fluorescence*/*blank fluorescence*) (1)

An anti-adhesion index (AAI) was used to express the anti-adhesion abilities of human urine and was calculated using Equation (2).

*AAI* = *adhesion inhibition* + 100 (2)

The blank AAI was set at 100 by assuming no anti-adhesion ability of synthetic human urine against P-type *E. coli*. The average AAI of two pre-consumption samples collected from a participant was considered her inherent AAI. UTI susceptibility was defined as the 50th percentile of inherent AAI. People with an inherent AAI lower than the 50th percentile were classified as UTI-susceptible, and those with an inherent AAI higher than the 50th percentile were UTI-nonsusceptible.

The responses to the cranberry juice were determined by AAI changes from baseline using Equation (3).

*AAI change* = *post*-*consumption AAI*—*baseline AAI* (3)

Participants with positive changes after cranberry juice intake were classified as positive responders to cranberry juice. Participants with negligible (changes below 10) or negative changes in AAI were classified as non-positive responders to cranberry juice.

### 2.9. Untargeted Urine Metabolomics on Uhplc-Q-Orbitrap-Hrms

To investigate the urinary metabolome in participants with low and high inherent AAI, 36 baseline urine samples were selected: 18 with high baseline AAI (133–164) and 18 with low baseline AAI (80–128). These samples were analyzed on a Thermo Q-Exactive Orbitrap mass spectrometer with Vanquish UHPLC and autosampler (Thermo Fisher Scientific, Waltham, MA, USA). Before the analysis, 50 µL of each urine sample was mixed with 20 µL of internal standard mix and 400 µL of 98:2 acetonitrile/water with 0.1% sodium azide. After centrifugal filtration, 400 µL of supernatant was dried and reconstituted in 50 µL of water containing 0.1% formic acid. The internal standard mix contained creatine-D_3_ H_2_O (methyl-D_3_), caffeine-D_3_ (1-methyl-D_3_), L-tryptophan-2,3,3-D_3_, and succinic acid-2,2,3,3-D_4_ (CDN Isotopes, Pointe-Claire, QC, Canada), salicylic acid-D_6_ (Millipore Sigma, Burlington, MA, USA)_,_ L-phenylalanine-^13^C_6_, propionic acid-^13^C_3_, and L-tyrosine ring-^13^C_6_, and L-leucine-^13^C_6_ (Cambridge Isotope Lab, Tewksbury, MA, USA). Solvent blanks and extraction blanks were analyzed with samples to monitor the validity and stability of data acquisition. The samples were randomized and analyzed in a single batch.

Chromatographic separation was achieved on an Avantor ACE Excel 2 C18-PFP UHPLC column (100 × 2.1 mm, 2.0 µm, VWR, Radnor, PA, USA) with 0.1% formic acid in water (mobile phase A) and 100% acetonitrile (mobile phase B). The flow rate was 350 µL/min with a column temperature of 25 °C. The gradient was as follows: 0–3 min, 100% A isocratic; 3–13 min, 0–80% B linear; 13–16 min, 80% B isocratic; 16–16.5 min, 80–0% B linear; followed by 3 min of re-equilibration of the column before each run.

A sample volume of 4 µL was injected for negative ionization mode and 2 µL for positive ionization mode. Full-scan mass spectra were acquired over a *m*/*z* range of 70–1000 with a high resolution of 70,000 at 200 *m*/*z*. Separate injections for MS^2^ scans were performed in a data-dependent MS/MS mode, selecting the top five most intense precursor ions per cycle, with full-scan mass resolution reduced to 35,000 at *m*/*z* 200. Heated electrospray ionization was employed with the parameters: sheath gas flow rate 45 arbitrary units (arb), auxiliary gas flow 10 arb, sweep gas flow 1 arb, spray voltage 35,000 V. The probe temperature was 350 °C. The capillary temperature was maintained at 320 °C. In-source collision-induced dissociation energy was 2 eV.

Raw data were converted to mzXML and imported into MZmine 2.53, which was used to perform mass detection, chromatogram building, smoothing, convolution, and deisotoping. The aligned features were gap-filled to account for any that the first alignment algorithm may have missed. The features were filtered to the minimum number of peaks per row. A list of the final features and their peak heights was exported for downstream multivariate analysis.

### 2.10. Statistical and Multivariate Analysis

The ratio of positive responders between UTI-susceptible and non-susceptible participants was compared using the Chi-square test. A *p* < 0.05 was considered significant. Metabolomic data were analyzed using SIMCA (version 18.0, Sartorius Stedim Data Analytics AB, Umeå, Sweden). Peak intensities of detected metabolomic features were used as predictors in partial least squares discriminant analysis (PLS-DA), partial least squares (PLS) regression, orthogonal PLS-DA (OPLS-DA), and orthogonal PLS (OPLS) regression models. For PLS-DA and OPLS-DA, participants were classified into high- and low-baseline urinary anti-adhesion activity (AAI) groups. For PLS and OPLS regression, urinary AAI was analyzed as a continuous outcome variable. PLS-DA was used to identify metabolomic profiles that discriminated between groups, whereas PLS regression identified features associated with urinary AAI. Model validation was performed using 7-fold internal cross-validation. Variable influence on projection (VIP) was used to assess the importance of X variables in models. X variables with VIPs > 1.0 were selected as important features for the PLS models and subsequently analyzed in OPLS-DA and regression models. The final discriminant features were selected based on S-plots of OPLS models with covariances of |p [1]| ≥ 0.05 and correlation coefficients of |p(corr) [1]| ≥ 0.5. A 200-time permutation test was conducted on each model to test for overfitting.

### 2.11. Annotation of Discriminant Metabolites

Discriminant features obtained from the multivariate analysis were identified and annotated. Before annotating, UHPLC-HRMS raw data files with MS/MS data were converted to an mzML format in MSconvert. The mzML files were then imported into Sirius [19]. The *m*/*z* ratios and retention times of discriminant features were used to search for matched MS/MS spectra in Sirius. Sirius enables annotation by connecting to multiple databases, including the Human Metabolome Database (HMDB), KEGG, the Global Natural Products Social Molecular Networking, and PubChem. The most robust annotations were selected based on Sirius and CSI: FingerID scores. Identification levels were determined using published guidance [20].

## 3. Results and Discussion

### 3.1. Cranberry Juice Contained Anthocyanins, Flavonols, and Procyanidins

**Figure 2:**
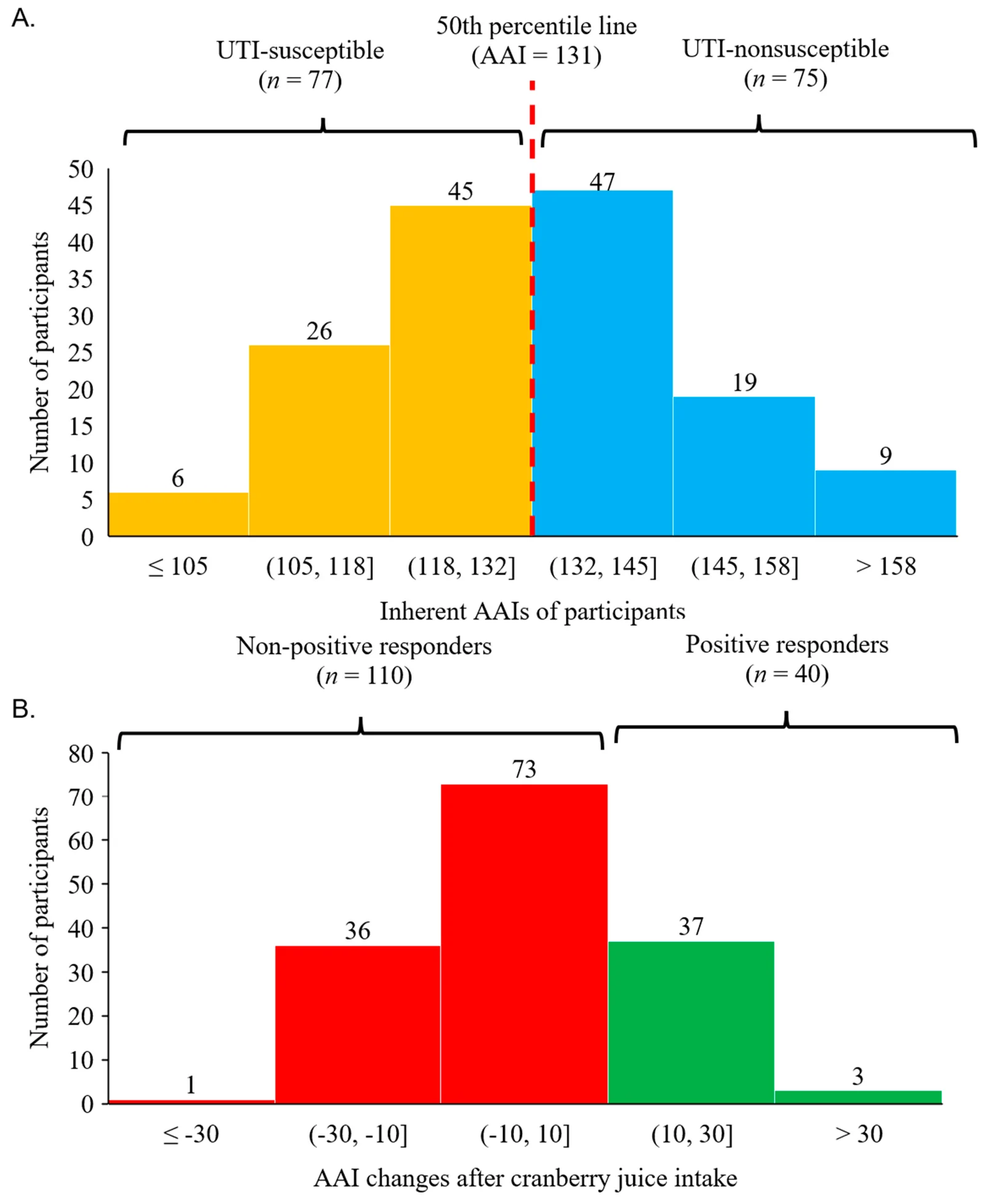
After the 10-day run-in period, 75 of 152 participants had baseline AAI above the 50th percentile and were classified as UTI-nonsusceptible. The other 77 participants were UTI-susceptible (**A**). After 4 days of cranberry juice intake, 40 of 150 participants (27%) had AAI changes above 10 and were classified as positive responders. The other participants were non-positive responders (**B**).

The polyphenol content of unsweetened pure cranberry juice is shown in Table 1. Anthocyanins were present as cyanidin-3-*O*-galactoside, cyanidin-3-*O*-arabinoside, peonidin-3-*O*-galactoside, and peonidin-3-*O*-arabinoside (Figure 2). Quercetin and myricetin were the major flavonols, whereas kaempferol and caffeic acid were not detected. The total amount of procyanidins in the cranberry juice was 30.25 ± 0.50 mg/100 mL based on the DMAC colorimetric assay. This proanthocyanin content is about 2.5 times that of a commercial juice cocktail, which typically contains 27% juice [21]. Ten ounces of the juice (296 mL) provided 89.5 mg of procyanidins per day. This dose was higher than 36 mg/day, which was suggested as a minimum effective dose required to trigger anti-adhesion activity in human urine [22].

### 3.2. Characteristics of Subjects

A total of 152 participants were enrolled and completed the 10-day run-in period and the collection of two baseline samples. Two participants withdrew from the study before the 4-day cranberry juice drinking period, and 150 of the original 152 participants completed the juice consumption. The flowchart is shown in Figure 1. Participants were aged 21 to 65 years, with the majority (about 56%) aged between 21 and 35 (Table 2). The 36–50 and 51–65 age groups accounted for approximately 21% and 23% of participants, respectively. Most individuals had a BMI within a normal range of 18.5–24.9 kg/m^2^. About 30% of participants had BMIs within the overweight range of 25–29.9 kg/m^2^, but not up to an obesity level. Races of participants included Caucasian (non-Hispanic or Latino, 59.9%), Asian (19.7%), African American (5.9%), Hispanic/Latino (12.5%), and American Indian (2.0%), with Caucasians being the predominant group.

### 3.3. Women’s Baseline Anti-Adhesion Capacities and Their Responses to Cranberry Juice Intake Varied Widely

The urinary anti-adhesion activities at baseline, considered inherent, were evaluated using an average value of 9 and 10 days for each participant. The baseline AAI among 152 participants showed a near-normal distribution, with values ranging from 84 to 170, as shown in the histogram in Figure 2A. Participants with low baseline AAI are susceptible to adhesion and invasion of UPEC onto the urinary tract and thus are considered susceptible to UTI. Whereas women with high baseline AAI are less susceptible. We chose a binary classification system using the 50th percentile of AAI (131) as the threshold for simplicity. Based on this system, seventy-five of 152 participants with inherent AAI above 131 were classified as UTI-nonsusceptible women. The other 77 participants who had lower inherent AAI were classified as UTI-susceptible with a higher risk of UTIs.

The P-type fimbriae on *E. coli* bind to the adhesin papG on the Galα1–4Gal core of glycolipids on the uroepithelial cell. Adhesion by P-type fimbriae is strong enough to counter the constant urine flow and lead to successful colonization in the urinary tract. Weakening such adhesion facilitates their removal by the urine flow [5].

Results suggest that urinary AAI is a normal aspect of human physiology and varies widely among healthy individuals. It is related to other aspects of human physiology, such as fasting glucose and blood pressure, which also vary widely, with 100 mg/dL and 120/80 mm Hg being used as thresholds for impaired fasting glucose and hypertension, respectively. Many factors contribute to such variation, including age, sex, and hormonal status; body size and composition; lifestyle factors; dietary and environmental influences; gut microbiome composition; and genetic differences.

The change in AAI from baseline among 150 participants after 4 days of cranberry juice intake showed a roughly normal distribution in the histogram in Figure 2B. Forty of 150 participants showed AAI changes above 10 and were classified as positive responders. The remaining 110 participants were classified as nonresponders. We chose 10 as a threshold because the coefficient of variation in the anti-adhesion assay was determined to be ≤10%.

**Figure 3:**
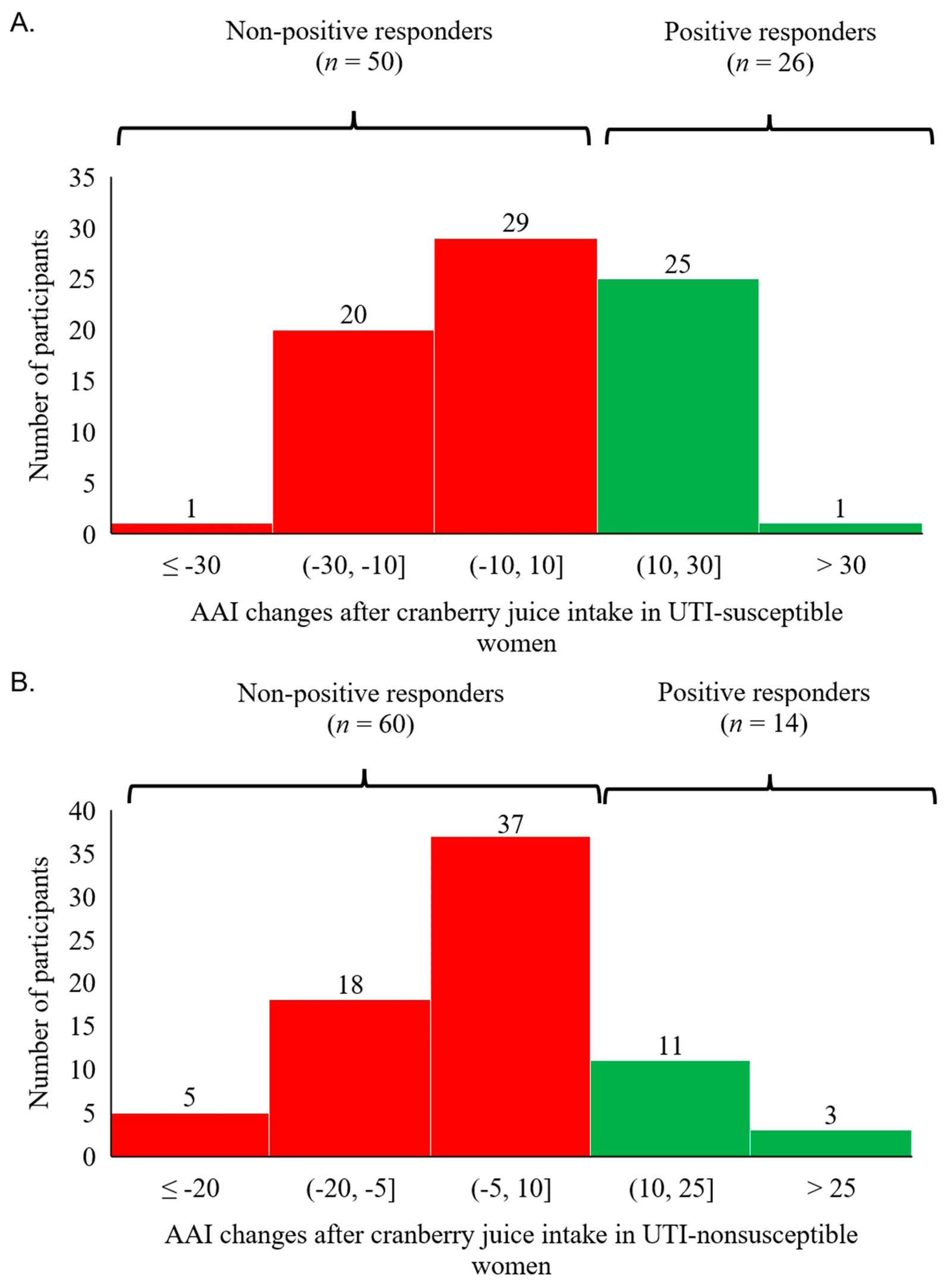
After 4-day cranberry juice intake, there were 34% positive responders and 66% non-positive responders in 76 UTI-susceptible women (**A**). Among the 74 UTI-nonsusceptible women, 19% were positive responders and 81% were non-positive responders (**B**).

The 76 UTI-susceptible participants were subdivided into 26 positive responders and 50 non-responders (Figure 3A). The 74 UTI non-susceptible participants consisted of 14 positive responders and 60 non-positive responders (Figure 3B). Both histograms showed a near-normal distribution. The percentage of positive responders in UTI-susceptible women was significantly higher than that in non-susceptible women (34.2% vs. 18.9%, χ^2^ test, *p* = 0.034).

The urinary AAI changes following cranberry intake have not been reported previously; however, they may be related to other human physiological responses, such as postprandial blood glucose. Zeevi et al. [23] provided identical meals to 800 healthy and prediabetic individuals and found high variability in postprandial blood glucose. Researchers attributed the variations to differences in gut microbiome, genetic and metabolic status, physical activity and sleep, and anthropometrics.

These data provide plausible explanations for the inconsistent results in the clinical trial investigating the use of cranberry juice to prevent UTIs. Women with potent inherent urinary antiadhesion activity are naturally resistant to UTIs and may not need cranberry juice or may benefit only slightly from it. Women with low inherent urinary AAI have a higher risk of UTIs; however, significant benefits may only be observed in those with strong positive responses. Previous trials did not account for variation in women’s inherent urinary AAI and changes after cranberry juice intake, which explains the high heterogeneity of their results. This study highlighted the importance of phenotyping participants prior to a randomized controlled trial to ensure reliable results.

A-type procyanidins in cranberries are presumed to be the bioactive compounds that confer anti-adhesion activity in urine [7]. The metabolic phenotypes of A-type procyanidins in humans are unknown, but multiple metabolic phenotypes have been reported for other polyphenols. For example, in adults, after consuming daidzein, 25–30% produce equol, and the rest are non-producers [24]. Humans can be stratified into three metabolic phenotypes following ingestion of ellagitannins and ellagic acid. About 25–80% of adults are urolithin A producers. About 10–50% of adults produce isourolithin A and/or urolithin B in addition to urolithin A. About 5–25% of participants did not produce any urolithins [25]. Variation in the gut microbiome was thought to be a major factor for multiple phenotypes.

### 3.4. Urinary Metabolome Differed in Participants with Low and High Baseline AAI

Urine AAI is directly affected by the urine metabolome. It is reasonable to postulate that urine with high AAI may contain higher levels of anti-adhesion metabolites than urine with lower AAI. To illustrate potential differences in the urine metabolome, metabolic features from the low-AAI and high-AAI groups were compared using OPLS-DA and regression models.

**Figure 4:**
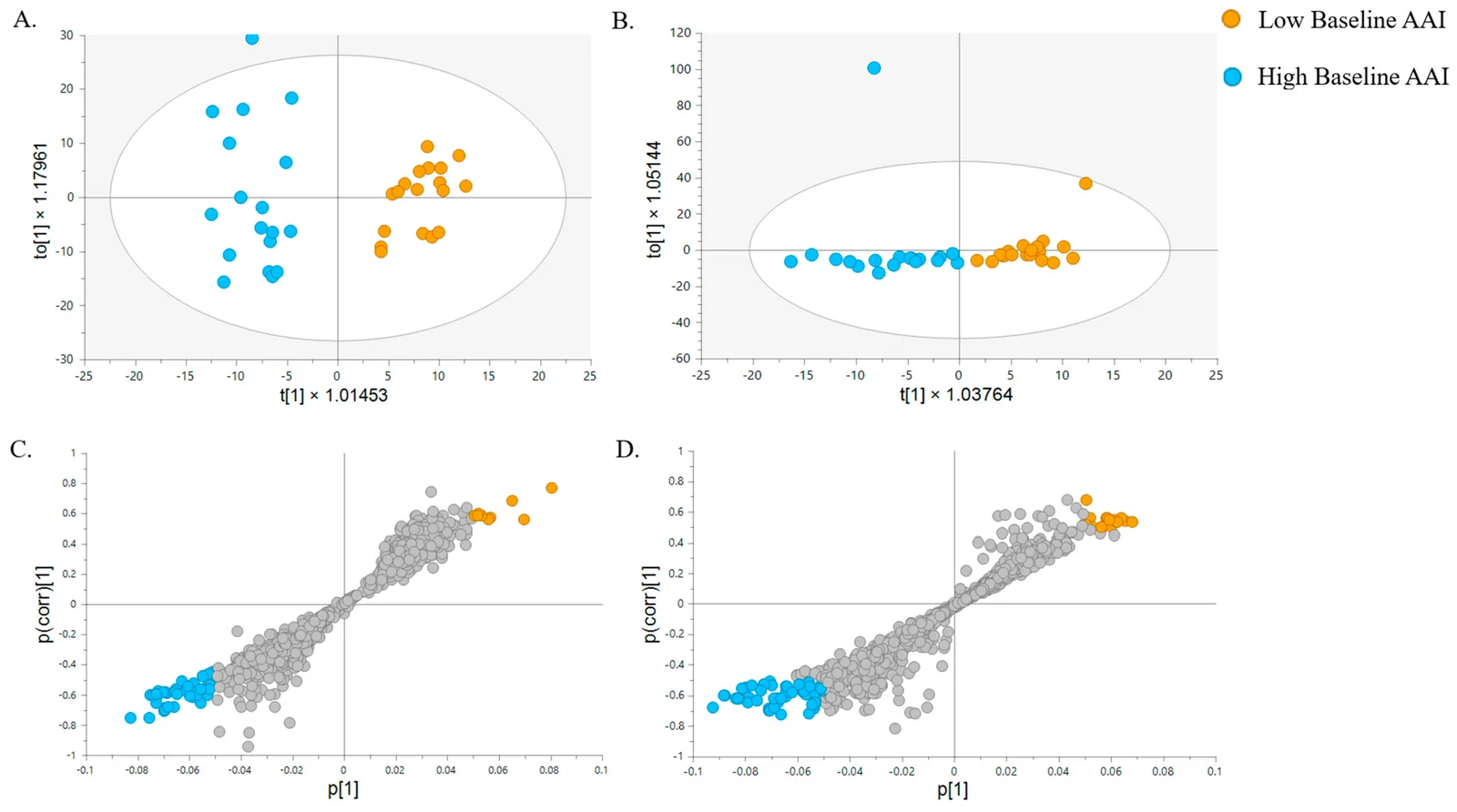
Score plots of OPLS-DA models (**A**,**B**) show separation of the urine metabolome between UTI-susceptible (*n* = 18, low baseline AAI) and nonsusceptible women (*n* = 18, high baseline AAI). S-plots (**C**,**D**) depict discriminant metabolic features in the urine that significantly contributed to the separation. Panels A and C: Plots for negative ionization. Panels B and D: Plots for positive ionization. Discriminant features that distinguished the two groups were marked on S-plots.

**Figure 5:**
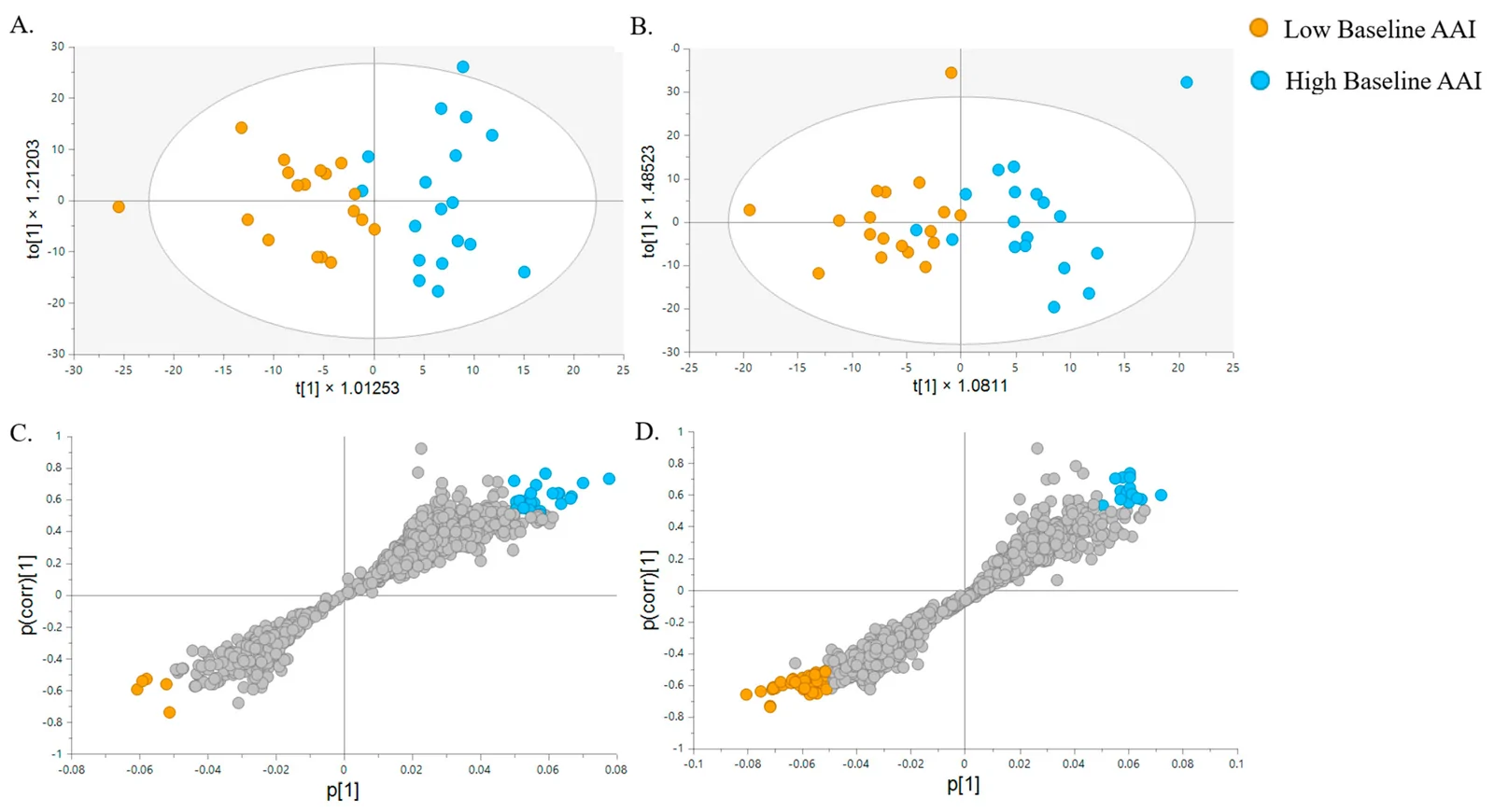
Score plots of OPLS regression models (**A**,**B**) show separation of the urine metabolome between UTI-susceptible (*n* = 18, low baseline AAI) and nonsusceptible women (*n* = 18, high baseline AAI). S-plots (**C**,**D**) depict discriminant metabolic features in the urine that significantly contributed to the separation. Panels (**A**,**C**): Plots for negative ionization. Panels (**B**,**D**): Plots for positive ionization. Discriminant features that distinguished the two groups were marked on S-plots. Table 3 shows the model parameters for the reverse-phase analysis. All the OPLS models consisted of one predictive and one orthogonal component. Cumulative R^2^X and R^2^Y values indicated the X and Y matrix variations explained by OPLS models. About 28% of X variation and 91% of Y variation for the negative ionization were explained by OPLS-DA and regression models. The OPLS-DA model explained about 54% of X variation and 77% of Y variation in the positive ionization data. Around 32% of X variation and 79% of Y variation in the positive ionization data were explained by the OPLS regression model. Cumulative Q^2^ indicated the models’ predictability. A 200-time permutation test was conducted for each reverse-phase analysis model, showing that Q2 values for full models were higher than those for reduced models (Supplemental Figure S1). Therefore, no overfitting was observed.

OPLS-DA score plots in Figure 4 show a clear separation between the low inherent AAI and high-AAI groups in both negative mode and positive mode, with groups (high inherent AAI vs. low inherent AAI) being the categorical Y responses. Figure 5 presents the score plots of the OPLS regression models, with the baseline AAI of the 36 samples as the numeric Y responses. Two samples with high inherent AAI overlapped with the low-AAI group, but the overall pattern shows good separation.

**Table 3:** OPLS-DA and regression models comparing low-AAI baseline urine samples (*n* = 18) with high-AAI baseline urine samples (*n* = 18) had acceptable goodness-of-fit (R^2^X, R^2^Y) and predictive ability (Q^2^).

Ionization	OPLS Models	Number of Components	R^2^X (cum) ^a^	R^2^Y (cum) ^a^	Q^2^ (cum) ^a^
Negative	OPLS-DA	1P ^b^ + 1O ^c^	0.286	0.915	0.816
	OPLS-regression	1P + 1O	0.283	0.918	0.738
Positive	OPLS-DA	1P + 1O	0.537	0.774	0.654
	OPLS-regression	1P + 1O	0.315	0.794	0.467

^a^ R^2^X (cum) and R^2^Y (cum) are the cumulative modeled variations in the X and Y matrices explained by OPLS models, respectively. Q^2^Y (cum) is the cumulative predicted variation in the Y matrix. ^b^ Predictive component. ^c^ Orthogonal component.

A total of 65 discriminant features in urine were selected based on S-plots from OPLS analysis of metabolites in the negative mode (Figure 4C and Figure 5C). Twelve discriminant features were associated with lower AAI, and 53 were associated with higher AAI. One hundred discriminant features were selected based on S-plots of OPLS analysis of metabolites in the positive mode (Figure 4D and Figure 5D): 61 were associated with higher AAI, and 39 were associated with lower AAI.

These data suggest that urine with low and high baseline AAI differ in their metabolomes. Some urine metabolites may have contributed to urinary anti-adhesion activity, while others may have reduced it. A previous study suggested that alterations in the urine metabolome by cranberry juice consumption are a potential mechanism for its UTI prevention functions [26]. However, this study did not consider participants’ metabolic phenotypes, which should be factored into future trials.

### 3.5. Annotation of Discriminant Urinary Metabolites Contributed to Inherent AAI

Five out of 65 discriminant features in negative ionization and seven out of 100 discriminant features in positive ionization were tentatively annotated using database searching. Six annotated metabolites correlated with high AAI, and the remaining five features correlated with low AAI (Table 4). Annotating compounds in untargeted metabolomics remains a major challenge. It is estimated that 90% of biological compounds are not covered by open mass spectral libraries. Accurate mass alone is insufficient for unambiguous identification because numerous compounds share similar or identical molecular formulas. Although high-resolution MS/MS data improve confidence, fragmentation spectra are highly dependent on instrument type, collision energy, ionization mode, and acquisition parameters, limiting the direct transferability of reference spectra across platforms. As a result, it is common to see that only 7–10% of features in untargeted metabolomics can be annotated [27].

Six annotated urinary metabolites correlated with higher inherent AAI. D-glucaro-1,4-lactone (HMDB0041862) belongs to a group of organic compounds called γ-butyrolactones containing a γ-butyrolactone moiety. It is formed in humans from D-glucaric acid and calcium-D-glucarate through the D-glucuronic acid pathway [28]. It is also found in various fruits and vegetables, such as oranges, apples, grapefruits, and cruciferous vegetables [29]. This compound has been detected in human blood and urine, but quantification has not been reported [30]. Some studies have found that D-glucaro-1,4-lactone inhibits lipid peroxidation in blood platelets through its antioxidant activity, which may help prevent cardiovascular diseases [31,32]. Moreover, it was found to positively affect liver carcinogenesis in rats by significantly lowering serum α-fetoprotein levels in a study [33].

TRIBOA β-D-glucoside (HMDB0341440) belongs to the class of organic compounds known as *O*-glycosyl compounds, typically found in cereals such as wheat, rye, and maize. It has been detected in human blood and urine [34]. 5-Acetylamino-6-formylamino-3-methyluracil (HMDB0011105) is an organic compound containing a hydroxyl group attached to a pyrimidine ring. It has been detected and quantified in human urine after caffeine intake [35].

(3,5-dihydroxyphenyl) Hydrogen sulfate is a phase-II metabolite of dietary polyphenols or microbiome-derived phenols. 3-Hydroxybenzoic acid-3-*O*-sulfate is a phenolic conjugate of 3-hydroxybenzoic acid and sulfate detected in human urine after tea intake [36].

3-Hydroxyisovaleryl carnitine is an acylcarnitine metabolite derived from branched-chain amino acid breakdown, particularly in the leucine catabolic pathway. Its increased concentrations in blood and urine have been considered biomarkers of organic acidemias and multiple carboxylase deficiency in newborns [37]. A recent study has shown that increased excretion of urinary 3-hydroxyisovaleryl carnitine was also associated with increased dietary leucine intake, an essential amino acid and potent stimulator of muscle protein synthesis [38]. Positive correlations were found between higher urinary 3-hydroxyisovaleryl carnitine concentrations and lower risk of all-cause mortality in kidney transplant recipients. 4-(2-Carboxyethenyl)phenyl β-D-glucopyranosiduronic acid is a phase-II metabolite of cinnamic acid-like phenols, which are abundant in plant-based foods [39].

Five annotated metabolites were significantly correlated with the low AAI group. (2R,2’S)-isobuteine (HMDB0030411), also known as *S*-(2-carboxypropyl) cysteine, was identified using both negative and positive ionization data. It belongs to the cysteine family and is found in onion-family vegetables. *N*-acetylalliin belongs to *N*-acyl-alpha amino acids, containing an alpha amino acid with an acyl group at its terminal nitrogen atom. It was found to be a metabolite in human serum and urine after consuming allium vegetables and cowpea [40,41]. *N*-acetylethionine sulfoxide is an *N*-acyl-alpha amino acid. It has been identified in human blood, urine, and feces [30,42]. *N*-acetyl-*S*-(2-carboxypropyl)cysteine and S-allylmercapturic acid have been identified as potential urinary biomarkers after consumption of allium vegetables, such as onions and garlic [43].

A strength of this study was the relatively large number of participants with diverse ethnic backgrounds. However, the age distribution was skewed toward the younger end, with 55.9% of participants in the 21–35 age group, reflecting the demographics of a college campus. This trial focused exclusively on P-type UPEC. Another limitation was that we did not record participants’ habitual dietary intake and physical activities during the trial. Cranberry juice consumption was limited to 4 days and thus did not include acute or chronic effects.

## 4. Conclusions

The present phenotyping trial evaluated the anti-adhesion capacity of baseline human urine and its changes after 4 days of cranberry juice consumption. There were considerable variations in baseline urinary anti-adhesion capacities among healthy women. The differential responses were likely influenced by variations in urinary metabolite profiles resulting from differences in individual metabolic pathways. Further investigation of the urine metabolome and the gut microbiome following cranberry intake is required to better understand the underlying mechanisms of individual differences in response. The novelty of this study was that it shows that inter-individual differences in intrinsic urinary anti-adhesion capacity and metabolomic profiles contribute to the variable intervention responses in previous clinical trials. It underscores the importance of properly phenotyping participants in randomized controlled trials to assess the efficacy of cranberries for UTI prevention. It suggested that women need personalized dietary advice on whether to use cranberry products to prevent UTIs.

## Figures and Tables

**Table 1:** Polyphenol content in unsweetened pure cranberry juice.

Phenolic Compounds		Concentration (mg/100 mL)
Anthocyanins	Cyanidin-3-*O*-galactoside	1.29 ± 0.01
	Cyanidin-3-*O*-glucoside	nd
	Cyanidin-3-*O*-arabinoside	0.659 ± 0.003
	Peonidin-3-*O*-galactoside	1.71 ± 0.01
	Peonidin-3-*O*-glucoside	nd
	Peonidin-3-*O*-arobinoside	0.575 ± 0.002
Flavonol	Myricetin	0.815 ± 0.010
	Quercetin	2.68 ± 0.04
	Kaempferol	nd
Procyanidin	Total procyanidins by DMAC assay	30.25 ± 0.50
Phenolic acid	Caffeic acid	nd

Data are mean ± standard deviation of triplicate tests. Caffeic acid, myricetin, quercetin, and kaempferol were quantified after cranberry juice polyphenols were hydrolyzed. nd, not detected.

**Table 2:** Demographics of participants in the metabolic phenotyping trial (*n* = 152).

Characterization	Group	Number of Participants	% of Participants
Age (year)	21–35	85	55.9
	36–50	32	21.1
	51–65	35	23.0
BMI (kg/m^2^)	18.5–24.9	107	70.4
	25–29.9	45	29.6
Race	Caucasian (non-Hispanic or Latino)	91	59.9
	Asian	30	19.7
	African American	9	5.9
	Caucasian (Hispanic/Latino)	19	12.5
	American Indian	3	2.0

**Table 4:** Annotated discriminant urinary metabolites based on |*p*| ≥ 0.05 and |p(corr)| ≥ 0.5 in OPLS-DA and regression models.

Retention Time (min)	*m*/*z*	Adduct	Model	p [1] ^a^	p(corr) [1] ^b^	Correlation ^c^	Annotation	Annotation Level
1.08	226.9966	[M+Cl]^−^	OPLS-regression	0.055	0.542	High AAI	D-glucaro-1,4-lactone	2
4.02	340.0677	[M-H_2_O-H]^−^	OPLS-DA	−0.053	−0.603	High AAI	TRIBOA β-D-glucoside	2
4.33	225.0632	[M-H]^−^	OPLS-regression	0.054	0.602	High AAI	5-Acetylamino-6-formylamino-3-methyluracil	2
7.06	246.0079	[M+C_2_H_3_N-H]^−^	OPLS-regression	0.051	0.588	High AAI	(3,5-dihydroxyphenyl) hydrogen sulfate	2
7.75	277.0027	[M+C_2_H_4_O_2_-H]^−^	OPLS-regression	0.063	0.638	High AAI	3-Hydroxybenzoic acid-3-O-sulphate	2
7.25	262.0378	[[M+H]^+^	OPLS-DA	−0.079	−0.645	High AAI	3-Hydroxyisovaleryl carnitine	2
7.50	373.1119	[M+CH_4_O+H]^+^	OPLS-DA	−0.057	−0.579	High AAI	4-(2-Carboxyethenyl) phenyl β-D-glucopyranosiduronic acid	2
3.61	206.0495	[M-H]^−^	OPLS-DA	0.053	0.588	Low AAI	(2R,2’S)-isobuteine	2
3.60	208.0634	[M+H]^+^	OPLS-regression	−0.071	−0.629	Low AAI	(2R,2’S)-isobuteine	2
6.22	220.0634	[M+H]^+^	OPLS-regression	−0.060	−0.555	Low AAI	N-acetylalliin	2
6.71	222.0791	[M+H]^+^	OPLS-regression	−0.050	−0.495	Low AAI	N-Acetylethionine sulfoxide	2
7.82	250.0739	[M+H]^+^	OPLS-regression	−0.058	−0.540	Low AAI	N-Acetyl-S-(2-carboxypropyl) cysteine	2
8.92	204.0686	[M+H]^+^	OPLS-regression	−0.070	−0.619	Low AAI	S-allylmercapturic acid	2

^a^ p [1] is a loading vector of covariance in the first predictive component. ^b^ p(corr) [1] is a loading vector of correlation in the first predictive component. Variables with |*p*| ≥ 0.05 and |p(corr)| ≥ 0.5 are considered statistically significant. ^c^ Correlations between urinary metabolites and AAI groups were determined based on p(corr) [1] in S-plots of OPLS models.

## Data Availability

The raw data supporting the conclusions of this article will be made available by the authors on request.

## Supplementary Materials

The following supporting information can be downloaded at: https://www.mdpi.com/article/10.3390/nu18193250/s1. Figure S1: Validation plots from 200 permutation tests for the reverse-phase analysis indicated the validity of the OPLS-DA and regression models. A, B) OPLS-DA and regression model validations for negative ionization, respectively. C, D) OPLS-DA and regression model validations for positive ionization, respectively.

## Abbreviations

AAI: Anti-adhesion index; arb, arbitrary unit; AU, absorbance unit; CFU, Colony-forming units; DMAC, Dimethylaminocinnamaldehyde; FI, Fluorescence intensity; HILIC, Hydrophilic Interaction Liquid Chromatography; HMDB, Human Metabolome Database; UHPLC, Ultra High-performance liquid chromatography; HRMS, High resolution mass spectrometry; MS^2^, Tandem mass spectrometry; *m*/*z*, Mass-to-charge ratio; OD, Optical density; OPLS, Orthogonal partial least squares; PLS-DA, partial least squares discriminant analysis; UTI, Urinary tract infection; UPEC, Uropathogenic *Escherichia coli*; VIP, Variable influence on projection.
