# Supplementary material for: Metabolic Phenotyping Identifies Distinct Urinary Anti-Adhesion Phenotypes and Differential Responses to Cranberry Juice Intake in Healthy Women
Source: Nutrients. 2026 Oct 1;18(19):3250. doi: 10.3390/nu18193250 (PMC13666710; doi:10.3390/nu18193250)
Supplement: Supplementary file 1 [file nutrients-18-03250-s001.zip › nutrients-4531718-supplementary.pdf]

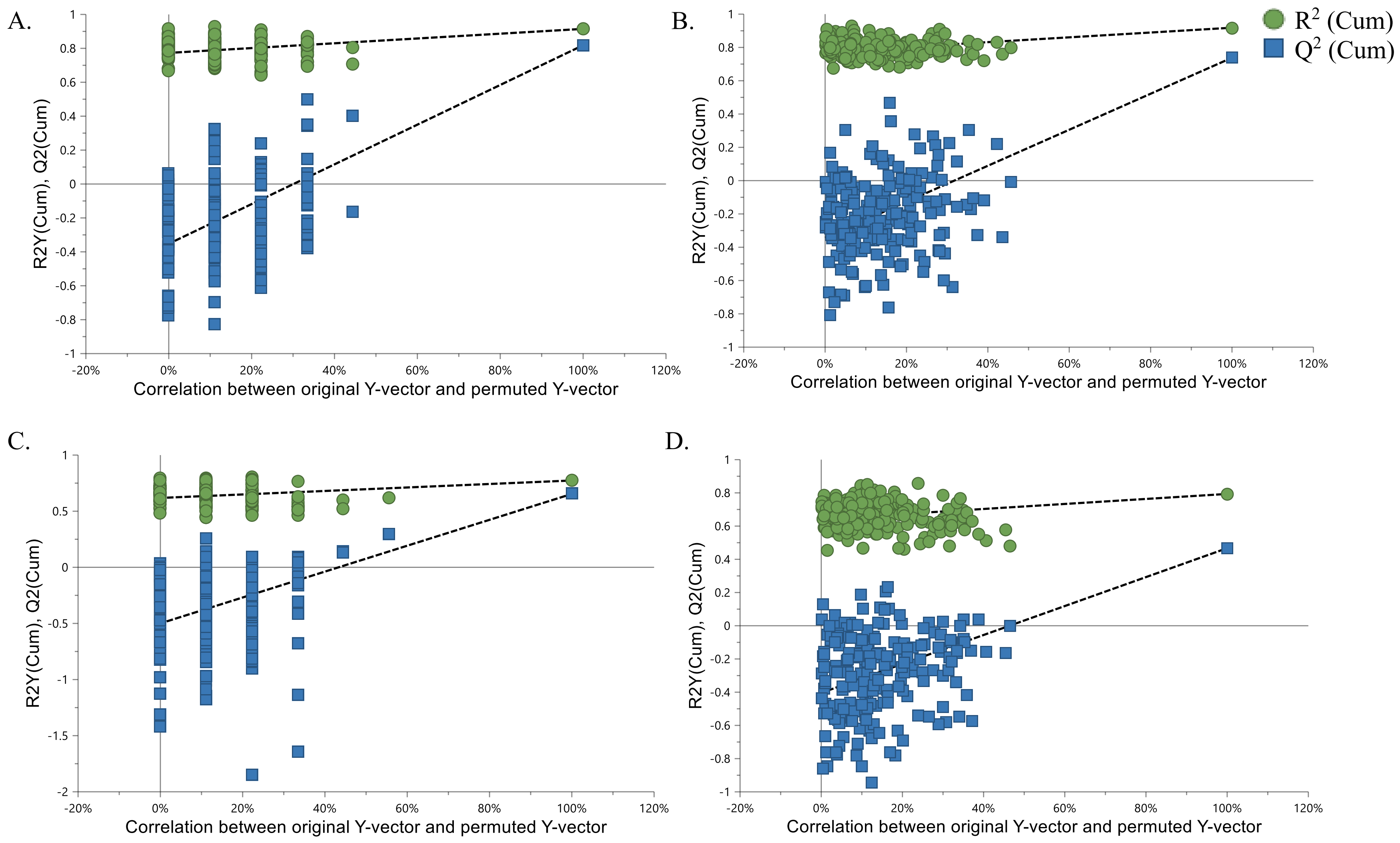

Figure S1. Validation plots from 200 permutation tests for the reverse-phase analysis indicated the validity of the OPLS-DA and regression models. (A, B) OPLS-DA and regression model validations for negative ionization, respectively. (C, D) OPLS-DA and regression model validations for positive ionization, respectively.
